# Chronic CD40L blockade is required for long-term cardiac allograft survival with a clinically relevant CTLA4-Ig dosing regimen

**DOI:** 10.3389/fimmu.2022.1060576

**Published:** 2022-12-08

**Authors:** Lukas W. Unger, Moritz Muckenhuber, Benedikt Mahr, Christoph Schwarz, Nina Pilat, Nicolas Granofszky, Heinz Regele, Thomas Wekerle

**Affiliations:** ^1^ Department of General Surgery, Division of Transplantation, Medical University of Vienna, Vienna, Austria; ^2^ Department of General Surgery, Division of Visceral Surgery, Medical University of Vienna, Vienna, Austria; ^3^ Clinical Institute of Pathology, Medical University of Vienna, Vienna, Austria

**Keywords:** costimulation blockade, CTLA4Ig, CD40L blockade, murine cardiac allotransplant, transplantation

## Abstract

**Introduction:**

In *de-novo* kidney transplantation, the CTLA4-Ig fusion protein belatacept is associated with improved graft function but also an increased risk of acute rejection compared to calcineurin inhibitor therapy. The combination with a second costimulation blocker could potentially improve outcome while avoiding calcineurin inhibitor toxicity. The aim of this study was to define the conditions under which the combination of CTLA4-Ig and CD40L blockade leads to rejection-free permanent graft survival in a stringent murine heart transplantation model.

**Methods:**

Naïve wild-type or CD40L (CD154) knock-out mice received a fully mismatched BALB/c cardiac allograft. Selected induction and maintenance protocols for CTLA4-Ig and blocking αCD40L monoclonal antibodies (mAB) were investigated. Graft survival, rejection severity and donor-specific antibody (DSA) formation were assessed during a 100-day follow-up period.

**Results and Discussion:**

Administering αCD40L mAb as monotherapy at the time of transplantation significantly prolonged heart allograft survival but did not further improve the outcome when given in addition to chronic CTLA4-Ig therapy (which prolongs graft survival to a median of 22 days). Likewise, chronic αCD40L mAb therapy (0.5mg) combined with perioperative CTLA4-Ig led to rejection in a proportion of mice and extensive histological damage, despite abrogating DSA formation. Only the permanent interruption of CD40-CD40L signaling by using CD40L^-/-^ recipient mice or by chronic αCD40L administration synergized with chronic CTLA4-Ig to achieve long-term allograft survival with preserved histological graft integrity in all recipients without DSA formation. The combination of α-CD40L and CTLA4-Ig works most effectively when both therapeutics are administered chronically.

Introduction

The advent of calcineurin inhibitors (CNI) in the 1980s established transplantation as the treatment of choice for end-stage organ failure in heart, lung, liver and kidney diseases. Subsequent improvements in surgical techniques and drug development resulted in an excellent short-term graft survival, while long term allograft survival in solid organ transplantation still remains unsatisfactory (1, 2). Apart from immune-mediated damage that contributes to chronic graft failure, CNIs exhibit direct nephrotoxicity (3). These adverse events, as well as an improved understanding of T cell activation have led to the conceptualized development of a new immunosuppressive drug class, namely costimulation blockers (4, 5). Belatacept, which is a fusion protein combining the mutated extracellular part of cytotoxic T-lymphocyte-associated protein 4 (CTLA4) with a human constant region fragment of IgG1, was intentionally designed to block the CD28 pathway and has been approved for kidney transplantation (6). Belatacept improved overall patient and graft survival (7), preserved kidney function better than cyclosporine A, and prevented *de novo* donor-specific IgG antibody formation more efficiently (8–10). However, belatacept is also associated with an increased risk of acute cellular rejection episodes, especially in the first year following transplantation (7, 11). Although these rejection episodes seemed to have no detrimental effect on long term outcome (10), they are nevertheless cause of concern and their avoidance through optimized drug combinations could decrease the number of hospitalization episodes early after transplantation.

In order to refine treatment strategies and test potential induction therapies, we have recently developed a murine vascularized heterotopic heart transplant regimen for chronic CTLA4-Ig monotherapy modeled after the clinically approved dosing regimen. In this model, C57BL/6 recipients of a fully mismatched BALB/c cardiac allograft rejected the transplanted heart with a median survival time (MST) of 60 days, with approximately 10 percent of the grafts surviving long-term (100 days) but showing extensive immune-mediated graft damage at the end of follow-up (12). Additionally, all recipients eventually developed donor specific antibodies (DSA) and showed early signs of acute rejection as early as 11 days after transplantation. We could demonstrate as proof-of-concept, that a 5-fold increase in dosing intensity (from 0.25mg to 1.25mg) significantly reduces, and a 25-fold increase (from 0.25mg to 6.25mg per time point) completely inhibits IgG DSA formation, leads to long-term graft survival and mostly abrogates histological signs of cellular rejection in all mice. In the clinic, however, an increase in dosing intensity by that order of magnitude is not an option (11). It is also very unlikely that a higher dose will be approved after the rate of acute rejection was slightly increased in the phase 3 trial with the more intense regimen (13).

Accordingly, other means are warranted to improve the efficacy of CTLA4-Ig. Apart from the costimulatory CD28-B7 signaling pathway, targeted by CTLA4-Ig, intercepting the interaction of CD40 and its ligand CD40L (CD154) has shown great potency in pre-clinical studies (14, 15). It is well established that the CD40-CD40L interaction is crucial for T cell activation, T cell-dependent antibody responses and immunoglobulin class switch, germinal center formation within lymph nodes, but also for memory development (16, 17). However, the clinical development of αCD40L monoclonal antibodies (mAB) had to be halted due to thromboembolic complications most likely resulting from Fc-receptor dependent platelet activation *via* immune-complexes (NCT02273960) (18, 19). Recent efforts to block CD40-CD40L signaling without platelet activation have resulted in the development of Fc-silenced αCD40L antibodies (20), and the additional development of αCD40 antibodies ASKP1240 (Bleselumab) and CFZ533. However, in a recent phase II trial, Bleselumab was associated with a greater incidence of biopsy-proven acute T-cell mediated rejection (TCMR) compared to a CNI-based regimen utilizing tacrolimus (21). Similar observations of limited efficacy were made in a clinical trial investigating CFZ533 in kidney transplantation resulting in early termination (NCT03663335). Partly, the reduced efficacy of αCD40 mAbs compared to αCD40L mAbs can be explained by the observation that CD11b can act as a second ligand for CD154, compensating for CD40 (22). However, these findings also highlight the complex interaction between distinct costimulatory signaling pathways that are potentially compensating for each other. Although blockade of both, CD28-B7 and CD40-CD40L alone is efficient, it has long been acknowledged that concomitant blockade of both pathways simultaneously is more effective than interfering with one alone. The original landmark study by Larsen et al. showed in a BALB/c to C3H setting that a short course of perioperative administration of CTLA4-Ig and αCD40L mAb led to heart allograft survival for more than 80 days and skin allograft survival of more than 50 days, while the respective monotherapies did not (14). In a subsequent paper in the more stringent and less costimulation-dependent BALB/c to C57BL/6 model, however, the combination of both drugs as induction resulted in rejection of skin allografts after a mean survival time (MST) of 56 days (23, 24), and no data on heart transplant survival in this setting was provided. In a study in rhesus monkeys, induction therapy of αCD40L mAb plus CTLA4-Ig alone significantly prolonged kidney allograft survival, but immune infiltrates were present even in functioning grafts and repeated administrations were required to halt acute rejection episodes (15). Thus, improved regimens achieving rejection-free long-term survival remain to be defined. Potentially, induction therapy at the time of transplantation with subsequent tapering/reduction to one of the two compounds would be an attractive approach. This approach could improve outcome and minimize adverse events, as death with a functioning graft significantly contributes to overall mortality (25, 26).

Thus, we set out to systematically test different combination regimens of CTLA4-Ig and αCD40L either as induction or chronic therapy to identify the most effective regimen in a fully mismatched, vascularized murine heart transplant (HTX) model.

## Materials and methods

### Mice

C57BL/6 and BALB/c mice were purchased from Charles River (Sulzfeld, Germany). Congenic B6.129S2-Cd40lg^tm1Imx^/J (CD40L^-/-^) mice were purchased from Jackson Laboratory (Maine, USA) and bred at the Center for Biomedical Research (Medical University of Vienna). All mice were housed in a protected environment and females were used between 12 and 16 weeks of age with a body weight between 20g and 25g. All animal experiments were approved by the internal review board of the Medical University of Vienna and by the Austrian Ministry of Science and Research (permission number GZ: BMWFW-66.009/0028-WF/V/3b/2015).

### Heterotopic heart transplantation

Cervical heterotopic cardiac transplantation was performed, as described previously (27). In brief, the recipient’s external jugular vein and common carotid artery were everted over a cuff under the microscope. Upon injection of 200IU heparin, the donor heart was harvested and flushed with 4 mL HTK solution (Custodiol, Koehler Chemie, Alsbach-Haenlien, Germany) through the aortic arch. The donor heart’s pulmonary trunk and ascending aorta were anastomosed over the prepared cuff with the recipient’s external jugular vein and common carotid artery respectively. Graft survival was assessed by visual inspection and palpation at least twice weekly for 100 days or until rejection. Rejection was defined as complete cessation of heartbeat and was confirmed in histological analysis.

### Cell culture

C57BL/6 splenocytes were isolated and cultured in RPMI 1640 medium (Biochrome, Berlin, Germany) supplemented with 10% FCS (Linaris, Dossenheim, Germany), PenStrep (100U Penicillin, 100μg Streptomycin/ml; Sigma), 10mM HEPES (MP Biomedicals), 1mM sodium pyruvate (Sigma), 1x non-essential amino acids (Sigma) and 10μM β-mercaptoethanol (Sigma). To assess CD40L and CD69 expression kinetics, 0.5x10^6^ splenocytes per well (96 well plate) were either stimulated with phorbol myristate acetate (PMA) (10ng/ml)/Ionomycin (0.2µg/ml), with 10μg/ml αCD3 (145-2C11) (BioXcell) and 1μg/ml αCD28 (37.51) (BioLegend) or with 0.5x10^6^ irradiated allogeneic donor cells (Balb/c) (for the mixed lymphocyte reaction, MLR). Early T-cell activation was assessed *via* CD69 surface expression. CFSE and Ki-67 were used to follow *in-vitro* proliferation of stimulated T-cells. To capture “cycling” CD40L, a surface mobilization assay was performed as described in (28). Briefly, the fluorescence-labeled anti-CD40L antibody was added directly into the medium during the last 4 hours of cell culture. In this way, both intracellular CD40L *cycling* to the cell surface during the incubation time and cell surface-expressed of CD40L were detected (*total* CD40L expression).

### Flow cytometry

Cells were stained for with Pacific Blue anti‐mouse CD3 (17A2), APC‐Cy7 anti‐mouse CD4 (RM4‐5) and anti-mouse CD25 (PC61), PE‐Cy7 anti‐mouse CD8 (53‐6.7), FITC anti-mouse Mac‐1 (M1/70), FITC anti‐mouse NK1.1 (PK136), PE anti-mouse CD11c (N418) and PE anti-mouse CD40L (SA047C3) were purchased from Biolegend. Cell labeling with carboxyfluorescein diacetate succinimidyl ester (CFSE) was performed with the Cell TraceTM CFSE Cell Proliferation Kit (Invitrogen, Carlsbad, CA). A BD FACS Canto II system (BD Bioscience, San Jose, CA, USA) was used for flow cytometry. Data was analyzed using the FlowJo software, version 10.0.8 (FlowJo LLC, Ashland, OR, USA).

### Dosing regimens

*Chronic* CTLA4-Ig treatment (Abatacept, Bristol-MyersSquibb, New York, USA) was administered according to the clinically approved dosing regimen (10 mg/kg body weight (7)), corresponding to 0.25mg/dose on days 0, + 4, +14, +28, +56, +84 (chronic CTLA4-Ig). For the indicated *CTLA4-Ig induction* group, 0.5mg were administered on days 0, 2, 4 and 6 (12). CTLA4-Ig/abatacept is used in mice since belatacept is not binding to murine CD80 and CD86.

Anti-CD40L (anti-CD154) monoclonal antibody (*αCD40L mAb*) *induction* (MR1, BioXCell, West Lebanon, USA) was administered on days 0 and +4 in a dose of 0.2mg/dose (low dose; LD) or 0.5mg/dose (high dose; HD). For the very high dose (VHD) anti-CD40L mAb induction group, 1mg/dose was administered on days -2, 0 and +4. For *chronic* α-CD40L mAb administration, 0.5mg were administered on days 0, + 4, +14, +28, +56, +84, or until cessation of heartbeat, respectively. All drugs were administered intraperitoneally in phosphate-buffered saline without Mg^2+^ and Ca^2+^ (PBS).

### Histology

Grafts were recovered at the end of the observation period. Samples were fixed in 7.5% formalin overnight and embedded in paraffin within 24h. Paraffin blocks were subsequently sectioned and stained with hematoxylin and eosin (H&E). Slides were scanned using an Aperio ScanScope scanner (Aperio Technologies, Inc., Vista, CA). Grading was performed according to the International Society for Heart and Lung Transplantation (ISHLT) 2004 guidelines (29) for cellular rejection score by an experienced pathologist (H.R.) blinded to the experimental background of tissue samples.

### Flow crossmatch

DSA were measured, as described previously (12). In brief, serum of recipient mice was obtained after rejection or at the end of follow-up. After heat inactivation, serum was incubated with donor‐ or recipient‐type thymocytes. Binding of IgG on thymocytes was measured by staining with anti–mouse IgG1‐FITC, IgG2a/2b‐FITC and IgG3-FITC (BD Biosciences) utilizing flow-cytometrical analysis on a BD CANTO II (BD Biosciences).

### Statistical analysis

Ordinal variables were compared using Fisher’s exact test. Metric values are presented as mean ( ± standard deviation). As values did not show normal distribution, a Mann–Whitney U test was used to compare groups. Survival curves were estimated according to the Kaplan–Meier method and compared with a log-rank test. GraphPad Prism version 8 (GraphPad Software, San Diego, California, USA) was used for statistical analysis. A p-value <0.05 was considered to denote statistical significance.

## Results

### CD40L is upregulated early upon stimulation *in-vitro*

**Figure 1:**
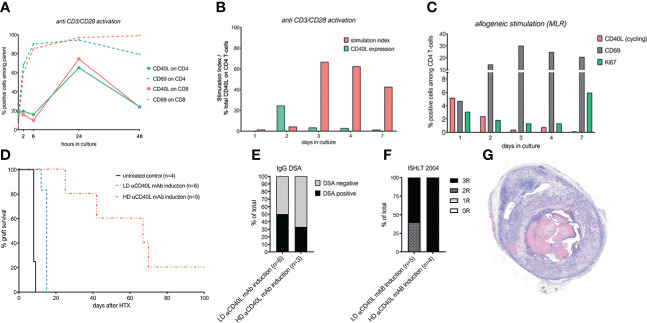
CD40L is upregulated early upon stimulation *in-vitro*, but its *in-vivo* blockade as induction alone does not facilitate long term cardiac allograft survival. **(A)** CD40L and CD69 surface expression on CD3^+^CD4^+^ and CD3^+^CD8^+^ T cells at indicated time points after stimulation with anti-CD3 and anti-CD28 mAb. **(B)** CD40L expression and stimulation index for CD3^+^CD4^+^ cells during activation with αCD3/αCD28 mAb. Stimulation index was defined as: %CFSE^dim^ cells within stimulated cells/%CFSE^dim^ cells within unstimulated control cells. **(C)** Mixed lymphocyte reaction of 0.5x10^6^ C57BL/6 splenocytes incubated with 0.5x10^6^ irradiated Balb/c splenocytes. CD69 and Ki67 are gated on CD3^+^CD4^+^ T-cells, CD40L expression on activated (CD69^+^) T-cells. **(D)** Cardiac allograft survival of untreated mice (n=4, black line), mice receiving low-dose αCD40L mAb induction (n=6, yellow dotted line) and mice receiving high-dose αCD40L mAb induction (n=5, 0.5mg; red dotted line). **(E)** Percentage of mice with detectable donor specific IgG antibodies at the time of rejection or end of long-term follow-up. In mice treated with HD αCD40L, sufficient amounts of serum were available only in 3/5 mice. **(F)** Severity of allograft rejection (ISHLT 2004 cellular rejection score) at the time of rejection or end of long term follow-up. **(G)** Representative histological section of the long term surviving heart in the HD αCD40L mAb induction group.

To assess CD40L expression kinetics in an *in-vitro* system that contains not only purified CD4^+^ T cells, we used unseparated C57BL/6 splenocytes, stimulated them with anti-CD3 and anti-CD28 mAbs and detected the dynamics of CD40L expression within the respective subpopulation using multicolor flow-cytometry. Adequate activation of the stimulated T-cells was confirmed by CD69 expression. Surface expression of CD40L was hardly detectable on naïve T-cells, but was rapidly upregulated in activated CD4 and CD8 T-cells and peaked after 24 hours upon polyclonal stimulation (**Figure 1A**). Similar kinetics were obtained in PMA/Ionomycin stimulated cells (**Supplementary Figure 1A**). However, the surface expression of CD40L is known to be highly dynamic due to internalization and rapid recruitment from lysosomes (28). Therefore, for subsequent cultures the staining antibody was added directly into the cell culture medium to capture surface as well as cycling CD40L on T-cells (total CD40L). To obtain further information on the relationship of CD40L expression and proliferation, we stimulated C57BL/6 splenocytes with αCD3/CD28 mAb and cultured them for 7 days. Again, total CD40L expression peaked early after stimulation, while proliferative activity, represented by the stimulation index, started only after 3 days when CD40L expression was already almost negligible (SI; %CFSE^dim^ cells within stimulated cells/%CFSE^dim^ cells within unstimulated control cells, **Figure 1B**). Finally, we stimulated C57BL/6 splenocytes with allogeneic irradiated BALB/c (donor) splenocytes in a classical 1:1 mixed lymphocyte reaction (MLR). Again, CD40L recruitment in CD4 T-cells peaked early upon activation within the first 2 days and then promptly declined thereafter (**Figure 1C**). T-cell activation steadily increased until day 3, while proliferative activity (Ki-67^+^) was not evident before day 7 of culturing.

Altogether, our *in vitro* assays showed that CD40L is rapidly expressed in T-cells within the first two days upon polyclonal and allogeneic stimulation, but sharply declines thereafter and precedes expansion, prompting us to test whether a short-term αCD40L mAb induction therapy is sufficient to induce long-term graft survival.

### α-CD40L mAb induction therapy alone does not induce long-term heart graft survival

Thus, we tested several αCD40L mAb induction regimens. Administering low dose (LD) αCD40L mAb induction (0.2 mg/dose on d0 and d4) increased the median survival time (MST) from 8 days in untreated controls to 15 days (p=0.0013). Increasing the dose of αCD40L mAb (0.5 mg/dose, HD) further increased the MST to 67 days, which is in line with previous reports (14) (**Figure 1D**). Both induction regimens reduced the incidence of DSA, but did not prevent DSA formation entirely (**Figure 1E**). The ISHLT-2004 scores of explanted hearts demonstrated extensive immune-mediated graft damage throughout both groups (**Figure 1F**), as well in the one long-term surviving heart (**Figure 1G**).

### Addition of αCD40L mAb induction therapy to chronic CTLA4-Ig treatment does not improve graft survival despite abrogation of DSA formation

**Figure 2:**
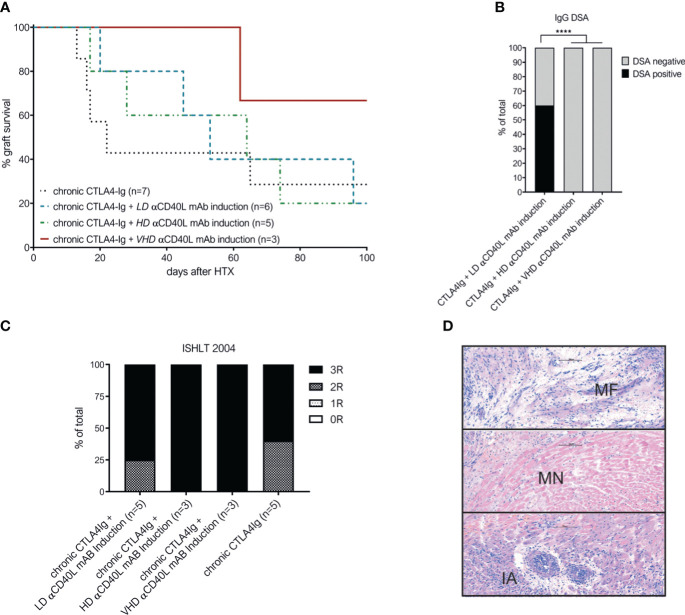
Combination of perioperative αCD40L mAb induction with chronic CTLA4-Ig administration does not significantly improve long-term outcome despite abrogation of DSA formation. **(A)** Addition of low-dose αCD40L mAb (n=6, turqouise interrupted line), high-dose αCD40L mAb (n=5, green line) or very high-dose αCD40L mAb (n=3, dark red dotted line) to chronic CTLA4-Ig therapy did not prolong heart allograft survival significantly compared to chronic CTLA4-Ig monotherpy (n=7; black dotted line). **(B)** Percentage of mice with detectable donor specific IgG antibodies (DSA) at the time of rejection or end of long-term follow up. **(C)** ISHLT-2004 scoring in histological slides of allografts harvested at rejection of end of follow-up. **(D)** Representative histological features of chronic allograft damage evident in long term surviving cardiac allografts: myocardial fibrosis (MF), miocardial necrosis (MN) and intimal arteritis (IA). **** indicates p<0.0001.

Next, increasing doses of αCD40L mAb induction therapy were added to the established dosing regimen of chronic CTLA4-Ig. Chronic CTLA4-Ig monotherapy demonstrated an MST of 22 days and long-term survival of 2/7 cardiac allografts. Additional induction with αCD40L mAb at low (LD), high (HD) or very high (VHD) dosages did not improve the median graft survival significantly (**Figure 2A**). DSA formation was abrogated in recipients treated with HD and VHD αCD40L induction (together with chronic CTLA4-Ig), but not in the LD αCD40L induction regimen demonstrating a dose-dependent effect (**Figure 2B**). Severe rejection was confirmed histologically in all grafts throughout all tested treatment regimens (**Figure 2C**). Some grafts survived long term, but features of chronic allograft damage including myocardial necrosis (MN), myocardial fibrosis (MF) and intimal arteritis (IA) were prominent (**Figure 2D**).

These results suggest that perioperative αCD40L mAb induction therapy is insufficient to improve allograft survival in addition to chronic administration of CTLA4-Ig.

### Recipient CD40L deficiency (CD40L^-/-^) or chronic αCD40L mAb therapy prevent DSA formation and cellular rejection in combination with chronic CTLA4-Ig

**Figure 3:**
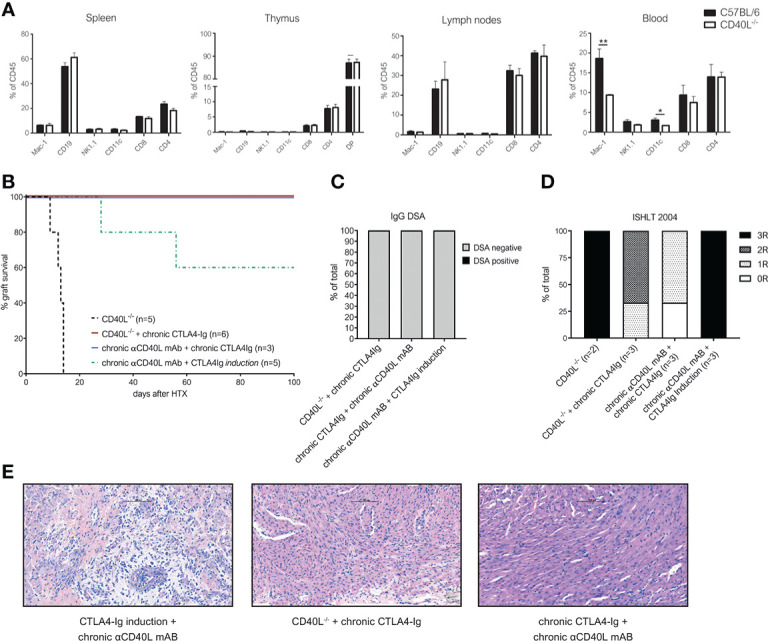
Chronic interception of both CD28-B7 and CD40-CD40L signaling leads to indefinite heart allograft survival, abrogates DSA formation and alleviates chronic rejection. **(A)** Distinct lymphocyte populations were analyzed in the spleen of naïve (C57BL/6) and CD40L knockout mice (CD40L^-/-^). Bars illustrate mean percentage of indicated leukocyte populations within CD45^+^ leukocytes + SD. **(B)** Cardiac allograft survival in CD40L^-/-^ recipients with (red line) or without (black dotted line) chronic CTLA4-Ig treatment and wild-type C57BL/6 recipients treated chronically with αCD40L and CTLA4-Ig (purple line) or chronic αCD40L and CTLA4-Ig induction (green dotted line). **(C)** Percentage of mice with detectable donor specific IgG antibodies (DSA) at the time of rejection or end of long-term follow up. **(D)** ISHLT-2004 grading of cardiac allograft tissue sections explanted at the end of follow up at day 100. **(E)** Representative histological images of H&E stained tissue sections from long term surviving hearts of recipients treated with CTLA4-Ig induction and chronic anti-CD40L (left), chronic CTLA4-Ig in CD40L^-/-^ mice (midle) and chronic CTLA4-Ig and anti-CD40L in wild-type mice (right). * indicates p<0.05; ** indicates p<0.01.

To test whether the complete absence of CD40-CD40L signaling in combination with chronic CTLA4-Ig prevents DSA formation and rejection, we used CD40L knockout mice (CD40L^-/-^, on a C57BL/6 background) as recipients. Previously it was reported that transplantation of BALB/c into CD40L^-/-^ mice leads to long-term heart graft survival when donor hearts were transplanted intraperitoneally, however significant graft injury was observed (30). In contrast to this report, we observed rapid rejection of cervically transplanted BALB/c heart allografts in CD40L^-/-^ mice with a median survival time of 13 days. On immunophenotypic analysis, we found no differences in leukocyte composition in lymph nodes, spleen or thymus, but a slightly decreased number of peripheral blood CD11c^+^ dendritic and Mac1^+^ myeloid cells in CD40L^-/-^ mice compared to wild type C57BL/6 (**Figure 3A**). These observations were consistent with original phenotyping results in CD40L^-/-^ mice (31). When we treated CD40L^-/-^ recipient mice with chronic CTLA4-Ig therapy, we observed indefinite graft survival in all mice (**Figure 3B**). To test whether similar results could be achieved with chronic pharmacological blockade of both costimulatory pathways, we administered αCD40L mAb in combination with CTLA4-Ig chronically, which again resulted in indefinite cardiac allograft survival in 3/3 mice (**Figure 3B**). In contrast, mice treated with only a short course of CTLA4-Ig induction and chronic αCD40L mAb rejected their hearts similar to chronic CTLA4-Ig monotherapy. All tested regimens abrogated the formation of IgG DSA (**Figure 3C**). Histologically, the long-term surviving hearts in the CTLA4-Ig induction plus chronic αCD40L group demonstrated extensive damage and consequently high ISHLT-2004 scores. Intercepting both costimulatory signaling pathways in CD40L^-/-^ plus chronic CTLA4-Ig, or chronic αCD40L plus chronic CTLA4-Ig in wild type recipients demonstrated adequate preservation of cardiac allografts integrity in the histological analysis 100 days after transplantation with median ISHLT-2004 scores of 2 and 1, respectively (**Figures 3D, E**).

Thus, we conclude that the permanent simultaneous blockade of both the CD28 and the CD40L pathway are necessary to maintain long-term graft survival without immune-mediated injury.

## Discussion

CD28-B7 and CD40-CD40L are two most intensively studied costimulatory pathways in solid organ transplantation. Intercepting both signals at the same time has demonstrated synergistic effects and remarkable efficacy across various pre-clinical transplant models. However, contrary to early reports, in a costimulation-blockade susceptible strain combination (BALB/c to C3H) (14), the short-term blockade of both signals does not induce donor-specific tolerance in a more stringent strain combination (BALB/c to C57BL/6) (23). Therefore, chronic administration of costimulation blockers, similar to classical immunosuppressive medication, is necessary. Whether both, CD28-B7 and CD40-CD40L interactions, are required to be blocked chronically for optimal outcomes had not been investigated in detail to date. Kirk et al. administered induction therapy for 14 days in a subgroup of their study of non-human primates and repeated administration at the time of rejection to achieve long-term graft survival, but ultimately reported immune cell infiltrates in the final histology without specification of histological rejection severity (15). Whether these identified lymphocytes are tolerogenic or a sign of subclinical rejection remains unanswered. Our findings indicate that over time even subclinical (T cell mediated) rejection even in the absence of donor-specific antibodies results in cumulative damage. Moreover, while pre-clinical models mostly focus on rejection as main outcome, in clinical practice more than 20% of kidney allografts are lost due to infections and malignancies associated with over-immunosuppression (26). Therefore, rational strategies to minimize the immunosuppressive burden, as much as reasonably possible, are an unmet clinical need.

Our *in-vitro* data demonstrates that CD40L is upregulated early upon T-cell stimulation, prior to proliferation, and promptly declines thereafter. Based on this observation, we hypothesized that a perioperative blockade of CD40L, as induction therapy, might synergize with chronic CTLA4-Ig treatment to prevent rejection in our murine heart transplantation model. Induction with αCD40L mAb on its own demonstrated dose-dependent efficacy and significantly prolonged cardiac allograft survival. However, the combination of αCD40L induction with our established chronic CTLA4-Ig regimen (modeled after the clinically approved dosing) was not sufficient to prevent rejection in this stringent, relatively costimulation blockade-resistant BALB/c to C57BL/6 heart transplant model. Within the 100 days of follow-up, most grafts were lost and the few long-term surviving hearts showed excessive damage in the subsequent histological analysis. Only the permanent absence of both CD40-CD40L and CD28-B7 signaling through a genetic CD40L knockout in recipients or a pharmacological CD40L blockade combined with chronic CTLA4-Ig led to long-term heart allograft survival with minimal immune-mediated graft damage. Our findings highlight again the potency of dual costimulation blockade, but also the need for further investigation of the mechanisms behind the “partial efficacy” and observed costimulation blockade resistant rejection. In this context, regulatory T-cells (Tregs) represent an attractive therapeutic target as their *in-vivo* expansion with IL-2 complexes (that allow for a more targeted delivery of IL-2 to Tregs than conventional IL-2) successfully prevented rejection under CTLA4-Ig in murine cardiac transplantation (32). The biggest advantage of combined costimulatory pathway blockade including CD40-CD40L is certainly the superior control over humoral allo-immunity, as CD40-CD40L is the main signaling pathway responsible for B cell activation and immunoglobulin class switch (33). Currently, in more than 20% of patients, DSA are the primary cause for graft loss (26) and improved immunosuppressive regimens preventing DSA formation are therefore warranted. In several clinical reports, belatacept was associated with a lower incidence of *de-novo* IgG DSA compared to CNI based regimens (8, 9, 34). In our model, all recipients receiving CTLA4-Ig monotherapy eventually developed IgG DSA. Addition of high dose and very high dose αCD40L induction, as well as chronic CD40L blockade (or genetic knockout) prevented IgG DSA formation entirely. This indicates that blocking CD40L might even be more efficient than CTLA4-Ig in terms of DSA prevention. Importantly, our study is limited to CD40L blockade together with CTLA4-Ig and did not investigate CD40 antagonists. Firstly, this is due to the fact that blocking antibodies specific for murine CD40 often times show partial agonistic effects (35), and currently no purely antagonistic anti-CD40 clone is commercially available to the best of our knowledge. Secondly, CD40L blockade has already been demonstrated to be superior to CD40 blockade in murine transplant models due to CD11b partially compensating for CD40 as ligand for CD40L (22). Thus, non-human primate studies investigating combined treatment regimens with CTLA4-Ig (Belatacept) and specific αCD40L clones that have been developed for clinical translation are warranted to compare their therapeutic potential, especially as more compounds targeting CD40L reach clinical evaluation (20, 36). Taken together, our work addresses open issues that remained for combined costimulation blockade with αCD40L and CTLA4-Ig. Herein we demonstrate in a robust and relatively costimulation blockade resistant heart transplant model that chronic blockade of both costimulatory signals is necessary to prevent T-cell mediated rejection and abrogate DSA formation.

## Data Availability

The raw data supporting the conclusions of this article will be made available by the authors, without undue reservation.

## Abbreviations

CFSE, carboxyfluorescein diacetate succinimidyl ester; CNI, calcineurin inhibitors; CTLA4, cytotoxic T-lymphocyte-associated protein 4; DSA, donor specific antibodies; HD, High dose; H&E, hematoxylin and eosin; HTX, heart transplantation; Ig, Immunoglobulin; ISHLT, International Society for Heart and Lung Transplantation; KO, Knockout; mAb, monoclonal antibody; MLR, mixed lymphocyte reaction; MMF, mycophenolate mofetil; PBS, Phosphate buffered saline; VHD, very high dose; HD, high dose; LD, low dose; MST, median survival time; PMA, phorbol myristate acetate; SPF, specific pathogen free; WT, wild-type.
