# Supplementary material for: Chronic CD40L blockade is required for long-term cardiac allograft survival with a clinically relevant CTLA4-Ig dosing regimen
Source: Front Immunol. 2022 Dec 8;13:1060576. doi: 10.3389/fimmu.2022.1060576 (PMC9773869; doi:10.3389/fimmu.2022.1060576)
Supplement: Supplementary file 1 [file DataSheet_1.docx]

**Supplementary Figure 1**


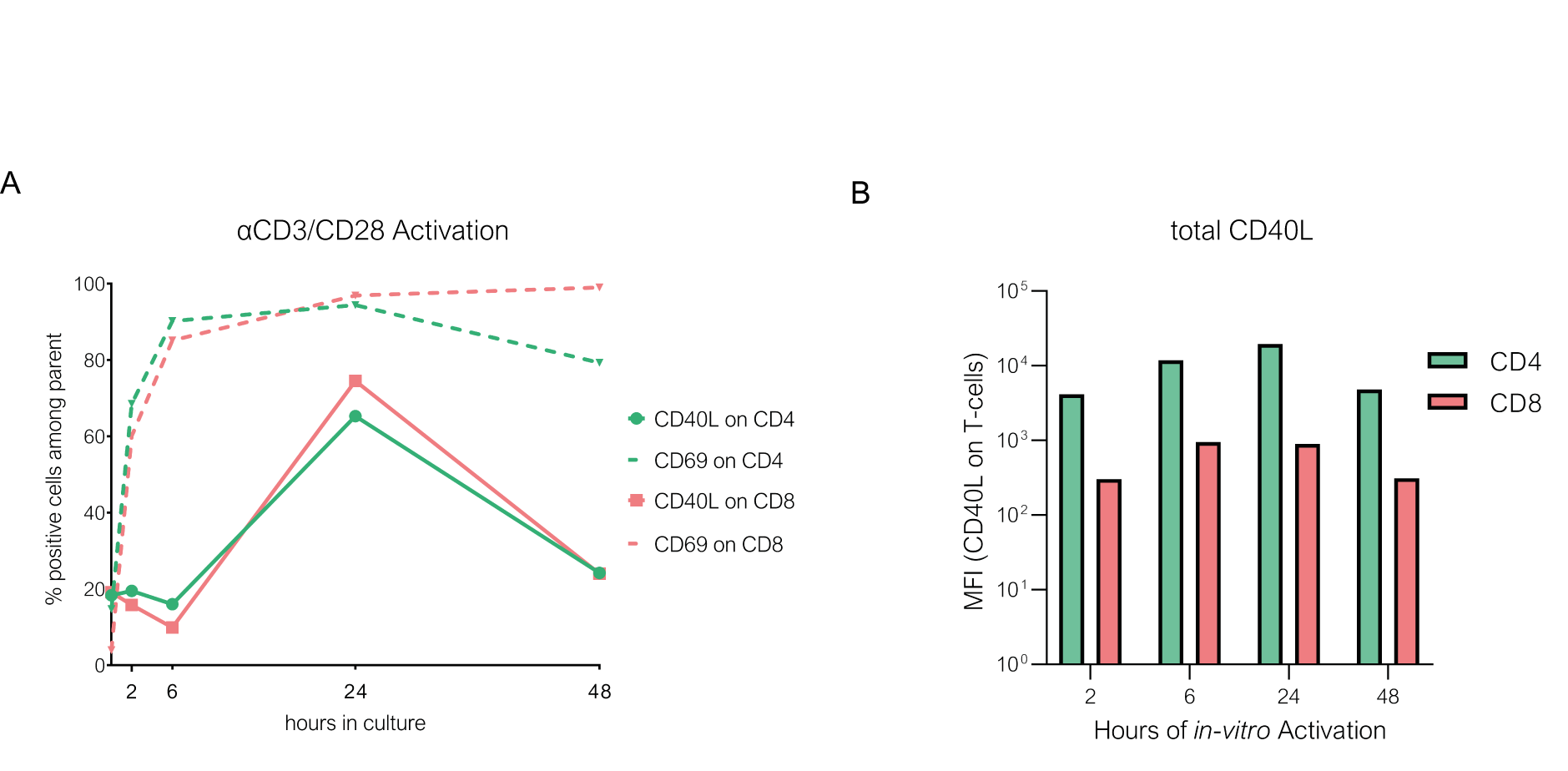


**Supplementary figure 1. CD40L expression upon stimulation *in-vitro using PMA/ionomycin***.

**(A)** CD40L and CD69 surface expression on CD3^+^CD4^+^ and CD3^+^CD8^+^ T cells at indicated time points after stimulation with PMA/ionomycin. **(B)** Total CD40L expression for CD3^+^CD4^+^ and CD3^+^CD8^+^ T cells during activation with PMA/ionomycin.
